# Influence of preexisting cognitive impairment and comorbidities on post-stroke outcomes: Dijon Stroke Registry

**DOI:** 10.1007/s00415-026-13782-5

**Published:** 2026-04-01

**Authors:** Sarah Kassir, Gauthier Duloquin, Yannick Béjot

**Affiliations:** Present Address: Université Bourgogne Europe, CHU Dijon Bourgogne, Service de Neurologie, Dijon Stroke Registry, PEC2 UR7460 Dijon, France

**Keywords:** Stroke, Epidemiology, Population-based study, Outcome, Case-fatality, Cognitive impairment, Dementia, Comorbidities, Frailty

## Abstract

**Background:**

Poststroke outcomes remain highly variable, particularly in older and frailer patients with multiple comorbidities. This study aimed to evaluate the independent impact of prestroke cognitive status and comorbidities on one-year functional and vital outcomes.

**Methods:**

Stroke patients (> 50 years old) were prospectively identified from the population-based Dijon Stroke Registry, France (2017–2022). Prestroke cognitive status (normal cognition, mild cognitive impairment (MCI), and dementia) and comorbidities were collected. Functional outcome (modified Rankin Scale) and survival were evaluated at 1 year. Multivariable logistic and Cox regression models were used to assess the associations between cognitive status and patient outcomes.

**Results:**

Among 1445 included patients (mean age 78.9 ± 12.0 years; 53.4% women), 907 (62.8%) had no prestroke cognitive impairment, 241 (16.7%) had MCI, and 297 (20.5%) had dementia. Patients with preexisting cognitive impairment were older and had a higher prevalence of vascular risk factors and comorbidities. One-year case-fatality was 25.7% in cognitively unimpaired patients, 35.3% in MCI patients, and 59.3% in patients with dementia. A poor functional outcome was observed in 49.9%, 66.2%, and 81.4% of patients, respectively. After adjustment for comorbidities, both MCI (OR = 1.45; 95% CI 1.01–1.81, *p* = 0.04) and dementia (OR = 2.65; 95% CI 1.81–3.87, *p* < 0.001) were associated with unfavorable functional outcomes. Furthermore, dementia was associated with increased 1-year death (HR = 1.98; 95% CI 1.60–2.48, *p* < 0.001).

**Conclusions:**

Preexisting cognitive impairment negatively influenced patient outcomes after stroke. The observed associations were independent of comorbid conditions, highlighting the importance of considering prestroke cognitive status in the design and interpretation of interventional studies aiming to improve stroke outcomes.

**Supplementary Information:**

The online version contains supplementary material available at 10.1007/s00415-026-13782-5.

## Introduction

Despite advances in acute therapeutic strategies post-stroke outcomes remain highly variable. This heterogeneity is influenced by demographic shifts, with a rising age among stroke patients, leading to a frailer population with numerous comorbidities [1, 2]. Previous studies showed that preexisting neurocognitive impairment is associated with poorer survival and functional outcomes [3–9]. In clinical practice, the presence of cognitive impairment and additional comorbidities generally influences medical decision-making [10], particularly when evaluating the benefit–risk ratio of acute interventions [11]. However, current treatment guidelines are primarily derived from clinical trials involving younger, healthier, and more homogeneous populations [12], which limit their applicability to the complex reality of older, multimorbid patients. Whether poorer post-stroke outcomes are primarily driven by the cognitive status itself or by the associated comorbid conditions has not been established in the literature [4, 13–15]. Therefore, the main objective of this study was to evaluate the influence of preexisting cognitive impairment and multiple comorbidities on both functional and vital outcomes in a population-based cohort of patients older than 50 years who experienced acute stroke.

## Methods

### Study population and case-ascertainment procedures

This study was based on data from the Dijon Stroke Registry, the methodology of which has been described elsewhere [5, 16, 17]. Briefly, this ongoing geographically defined population-based registry complies with the quality criteria for conducting incidence stroke studies [18], and the guidelines for the reporting of incidence and prevalence studies in neuroepidemiology according to Standards of Reporting of Neurological Disorders (STROND) [19]. All cases of symptomatic stroke that occurred among the population of the city of Dijon, France (about 158,000 inhabitants) were identified thanks to multiple overlapping sources of information including: hospital admission and emergency department registers, including the Dijon University Hospital where the only stroke unit and department of neurology of the area is located, and private hospitals of the city; computerized hospital discharge diagnostic codes using the International Classification of Diseases, 10th revision (ICD-10) (I61, I62, I63, I64, G45, G46, and G81); computer-generated list of all requests for brain and cerebral vascular imaging in public and private centers; Direct transmission from general practitioners and private neurologists of the city; and death certificates to identify out-of-hospital deaths. Stroke was defined according to the World Health Organization (WHO) criteria [20], and was classified as ischemic stroke or spontaneous intracerebral hemorrhage. For the present analysis, we considered patients older than 50 years who experienced a stroke between January 1, 2017 and December 31, 2022.

### Data collection

For each patient, the following vascular risk factors and past medical history were collected: hypertension (high blood pressure recorded in medical history or patients under antihypertensive treatment), diabetes mellitus (glucose level ≥ 7.8 mmol/L reported in the medical record or patients taking insulin or oral hypoglycemic agents), hypercholesterolemia (total cholesterol level ≥ 5.7 mmol/L reported in the medical history or patients treated with lipid-lowering therapy), smoking status (current smoker, past smoker or never smoker), excessive alcohol consumption (defined as alcohol intake ≥ 2 units a day in women and ≥ 3 units a day in men), history of stroke or transient ischemic attack (TIA), coronary artery disease, peripheral artery disease, chronic heart failure, and atrial fibrillation (either previously known or diagnosed during stroke evaluation). Additional chronic conditions were collected and are fully detailed in Supplementary file. Variables were selected based on validated comorbidity scales such as the Charlson [21], and the Elixhauser [22] Comorbidity Indexes. Some items in the medical history were grouped into categories: coronary artery disease (including angina, coronary angioplasty/stenting and coronary artery bypass surgery, and myocardial infarction), peripheral vascular disease (including chronic obliterative arteriopathy of the lower limbs, lower limbs angioplasty/stenting and vascular surgery), cancer (including solid cancer and hemopathy), digestive tract disease (including liver diseases and digestive ulcer), musculoskeletal disease (including rheumatologic disease and femoral neck fracture), psychiatric disorder (including mood and psychotic disorders), and other systemic and autoimmune disease (including dysthyroidism). Previous medications were reported by class: antidiabetics, antihypertensive treatment, lipid-lowering therapy, anticoagulant, and antiplatelet agents.

Prior-to-stroke cognitive status was assessed based on interviews with patients, their relatives, and/or their general practitioner, as well as the review of medical files, as previously described [5, 16]. Investigators searched for overlapping sources of information, including primary care records, specialist consultation records, hospital outpatient clinic documentation, and hospital discharge summaries obtained from a unique electronic medical file. Cognitive status was adjudicated according to the fourth edition of the Diagnostic and Statistical Manual of Mental Disorders (DSM-IV) criteria, based on all available information. In cases of uncertainty, all relevant data were reviewed by the senior physician with expertise in stroke and dementia (YB). Patients were classified as follows: no cognitive impairment, mild cognitive impairment (MCI), defined as a cognitive decline without any interference with activities of daily living, or dementia, defined as a cognitive decline sufficient to interfere with independence in activities of daily living. Patients for whom it was impossible to obtain reliable information were considered as having missing data about prestroke cognitive status. Functional status before stroke was evaluated using the modified Rankin Scale (mRS) score. Stroke severity at onset was quantified using the National Institutes of Health Stroke Scale (NIHSS) score obtained at the first clinical examination. When this score was not reported in the medical file, it was estimated retrospectively on the basis of medical records and charts, as previously validated in the literature [23]. In patients with ischemic stroke, acute revascularization therapy (either IV thrombolysis and/or mechanical thrombectomy) was collected.

### Follow-up of patients and outcomes

Patients included in the Dijon Stroke Registry were followed up at 1 year as part of the Dijon Stroke Cohort (DISCO). Vital status was systematically recorded using the national French registry of deaths that provides an exhaustive list of deceased patients on a monthly basis. Information on vital status was available for all patients. In surviving patients, functional status was assessed during a telephone interview by a trained clinical research assistant or nurse. If the patient or their relatives could not be reached, information about mRS score was retrieved by reviewing the medical records if patients attended a 1-year follow-up consultation with a neurologist. A favorable outcome was defined as a mRS score of 0–2. In patients with a premorbid mRS score > 2, favorable outcome was defined as a return to their prestroke mRS score.

### Statistical analysis

For baseline characteristics, proportions, means, and medians were compared using the Chi-square test, and the Kruskal–Wallis test when appropriate. Person-days were calculated from the date of stroke onset until death, the last contact date, or the end of follow-up at 365 days. Survival curves according to cognitive status before stroke were obtained using Kaplan–Meier analysis. Cox regression models were used to evaluate the association between premorbid cognitive status and death at 1 year. In multivariable analyses, we first introduced age and sex. In subsequent models, we introduced baseline variables with a p value < 0.20 in the unadjusted models. A stepwise backward selection procedure was applied to obtain the final model. At each step, variables were sequentially removed and nested models were compared using likelihood ratio tests. Variables were retained in the final model if their removal significantly worsened the model fit (*p* < 0.05). Age, sex, and cognitive status were forced into the models. Logistic regression analyses were used to assess the association between premorbid cognitive status and poor functional outcome. In multivariable models we introduced age, sex, and variable with a *p* value < 0.20 in univariate analysis, and a stepwise backward selection procedure was applied to derive the final model, as described above. Sensitivity analyses were conducted among patients with ischemic stroke to account for the potential effect of acute revascularization therapy on 1-year survival and functional outcome. Cox proportional hazards and logistic regression models were fitted using the same procedure as described above, with additional adjustment for acute revascularization therapy as a confounding variable. *p* values < 0.05 were considered statistically significant. Statistical analysis was performed with STATA@13 software (StataCorp LP, College Station, TX, USA).

## Results

**Fig. 1:**
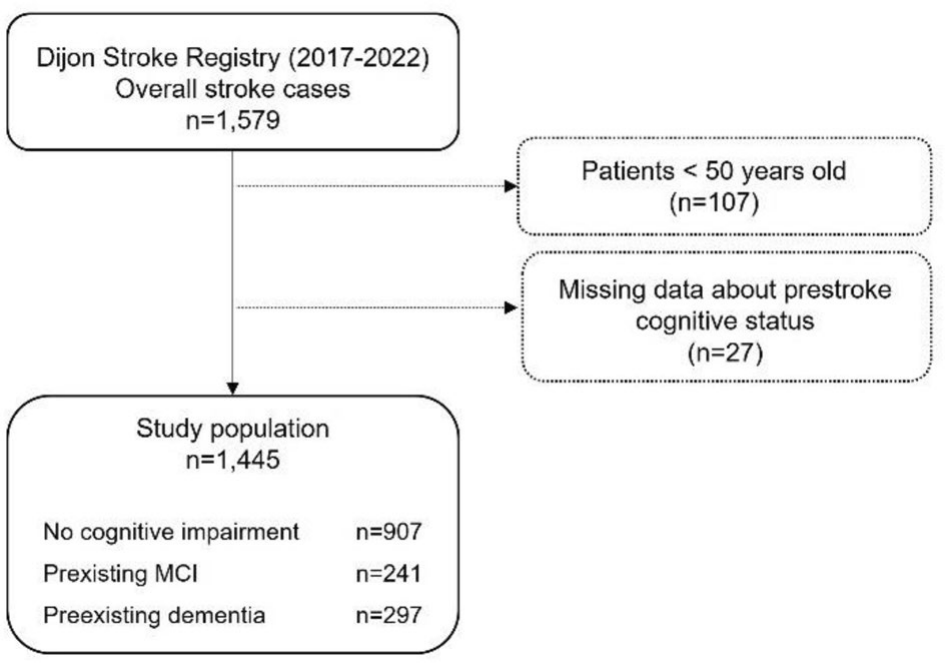
Study flowchart. *MCI* mild cognitive impairment

Over the study period, 1579 cases of acute ischemic and hemorrhagic stroke occurred in Dijon, France. After excluding patients younger than 50 years old (*n* = 107), and those with missing data for premorbid cognitive status (*n* = 27, 1.8%), 1445 patients were included (mean age ± SD: 78.9 ± 12.0; 53.4% women), including 907 (62.8%) patients without cognitive impairment, 241 (16.7%) patients with premorbid MCI, and 297 (20.5%) with prior dementia (Fig. 1).

**Fig. 2:**
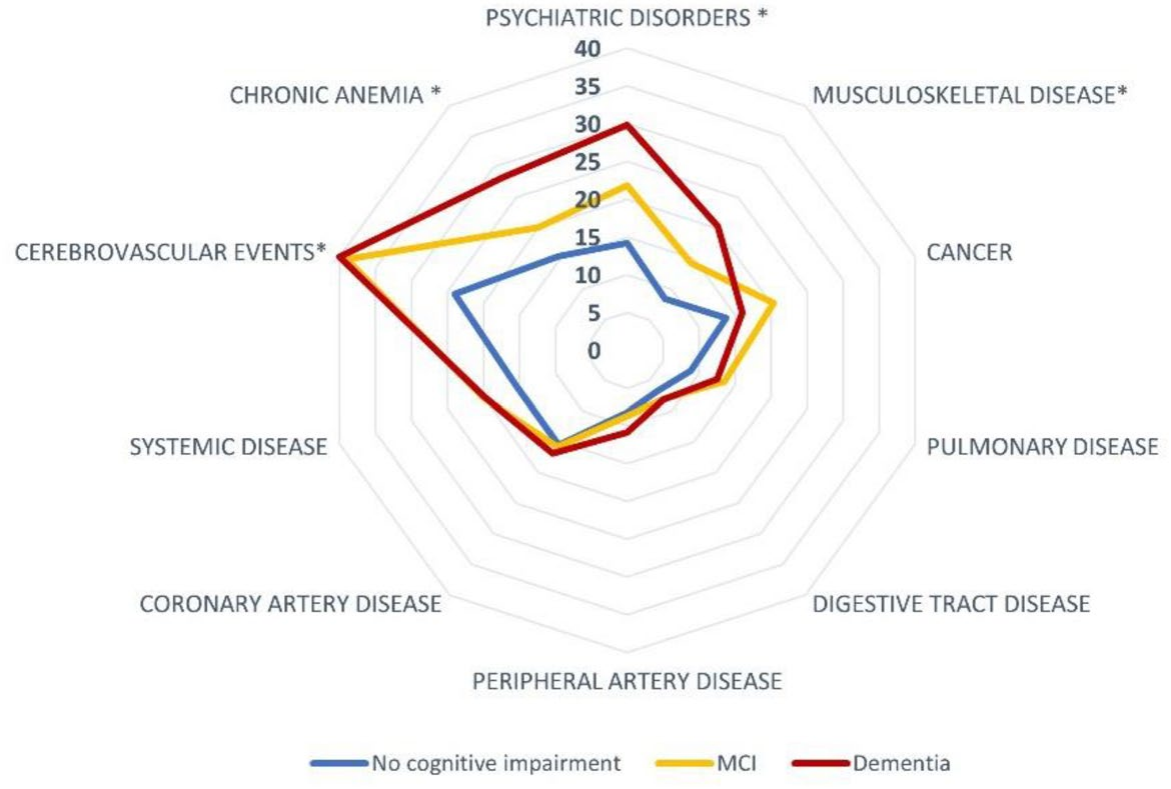
Distribution of comorbidities according to prestroke cognitive status. *MCI* mild cognitive impairment

Baseline characteristics of patients according to their premorbid cognitive status are shown in Table 1. Patients with preexisting cognitive impairment were older, more often women, and had greater prestroke disability. They differed from their cognitively unimpaired counterparts with regard to a higher prevalence of hypertension and atrial fibrillation. Stroke severity at onset was greater in cognitively impaired patients. In those with ischemic stroke, acute revascularization therapy was used less frequently than in patients without premorbid cognitive impairment. Regarding other comorbidities (Table 1 and Fig. 2), patients with cognitive impairment had more following conditions: cerebrovascular event history (*p* < 0.001 difference observed for previous stroke), chronic kidney failure, musculoskeletal disease (*p* < 0.001, difference observed for osteoporosis and femoral neck fracture), chronic anemia (*p* < 0.001), parkinsonism (*p* < 0.001), epilepsy (*p* = 0.002) and psychiatric disorders (*p* < 0.001, difference observed for psychotic and treated mood disorders), venous thromboembolic event (*p* = 0.02, difference observed for deep venous thrombosis), and hypothyroidism (*p* = 0.048).

**Table 1:** Baseline patients’ characteristics according to their prestroke cognitive status

	Prestroke cognitive status			*p* value
	No cognitive impairment (*n* = 907) *n*(%)	Mild cognitive impairment (*n* = 241) *n*(%)	Dementia (*n* = 297) *n*(%)	
Demographics				
Age, years, mean (± SD)	76 (± 12)	83 (± 10)	85 (± 8)	** < 0.001**
Age, years, median	76 [66–85]	85 [77–90]	86 [81–92]	** < 0.001**
Male sex	461 (50.9)	105 (43.6)	111 (37.4)	** < 0.001**
Vascular risk factors				
Hypertension	623 (68.7)	191 (79.3)	234 (78.8)	** < 0.001**
Diabetes mellitus	195 (21.6)	66 (27.6)	77 (26.0)	0.08
Hypercholesterolemia	381 (42.0)	103 (42.7)	113 (38.1)	0.43
Atrial fibrillation	268 (29.6)	94 (39.0)	119 (40.1)	** < 0.001**
Smoking status				** < 0.001**
Never smoker	488 (55.9)	145 (63.6)	219 (78.8)	
Current or past smoker	386 (44.1)	83 (36.4)	59 (21.2)	
Alcohol intake				0.23
No abusive alcohol intake	761 (87.5)	196 (87.5)	254 (91.4)	
Current or past alcohol intake	109 (12.5)	28 (12.5)	24 (8.7)	
Sleep apnea	61 (6.7)	19 (7.9)	23 (7.7)	0.74
Medical history				
Cerebrovascular event				
Previous TIA	95 (10.5)	37 (15.4)	34 (11.5)	0.11
Previous stroke	149 (16.4)	73 (30.3)	106 (35.7)	** < 0.001**
Peripheral artery disease				
Lower limb peripheral artery disease	65 (7.2)	18 (7.5)	32 (10.8)	0.13
Lower limb vascular surgery	16 (3.3)	4 (2.4)	2 (1.2)	0.39
Lower limb angioplasty	23 (4.7)	6 (4.3)	2 (1.2)	0.14
Coronary artery disease				
Myocardial infarction	103 (11.4)	30 (12.5)	38 (12.8)	0.76
Coronary artery bypass surgery	33 (6.7)	9 (6.3)	10 (6.2)	0.99
Coronary stenting	33 (6.8)	9 (6.3)	10 (6.2)	0.056
Valvular heart disease	108 (11.9)	42 (17.5)	55 (18.6)	**0.005**
Heart failure	48 (5.3)	20 (8.4)	28 (9,5)	**0.02**
Preserved cardiac function	32 (3.6)	13 (5.5)	20 (6,8)	0.054
Altered cardiac function	16 (1.8)	7 (3.0)	8 (2.7)	0.42
Cancer				
Solid cancer				0.29
No cancer history	728 (81.0)	181 (75.7)	241 (81.4)	
Recovered	81 (9.0)	41 (17.2)	33 (11.2)	
Active non metastatic	45 (5.0)	12 (5.0)	17 (5.7)	
Active with lymph-node metastasis	17 (1.9)	1 (0.4)	2 (0.7)	
Active with organic metastasis	28 (3.1)	4 (1.7)	3 (1.0)	
Hemopathy	36 (4.0)	5 (2.1)	11 (3.7)	0.37
Lymphoma	12 (1.3)	3 (1.3)	6 (2.0)	0.66
Chronic lymphocytic leukemia	5 (0.6)	1 (0.4)	1 (0.3)	0.89
Acute leukemia	3 (0.3)	1 (0.4)	1 (0.3)	0.97
Myeloma	6 (0.7)	0 (0.0)	1 (0.3)	0.39
Myeloproliferative syndrome	9 (1.0)	0 (0.0)	2 (0.7)	0.28
Chronic pulmonary disease	79 (8.8)	32 (13.5)	37 (12.5)	0.42
COPD	47 (5.2)	21 (8.8)	19 (6.4)	0.11
Asthma	30 (3.3)	9 (3.8)	14 (4.7)	0.54
Restrictive ventilatory disorder	6 (0.7)	4 (1.7)	5 (1.7)	< 0.001
Kidney function				** < 0.001**
Normal kidney function	515 (57.4)	92 (38.8)	110 (37.3)	
Clearance 60–90	254 (28.3)	90 (38.0)	108 (36.6)	
Clearance 30–60	92 (10.3)	40 (16.9)	59 (20.0)	
Clearance 15–30	27 (3.0)	14 (5.9)	16 (5.4)	
Clearance < 15	10 (1.0)	1 (0.4)	2 (0.7)	
Musculoskeletal disease				
Rheumatologic disease	50 (5.6)	15 (6.3)	42 (14.2)	** < 0.001**
Osteoporosis	30 (3.3)	14 (5.9)	39 (13.2)	** < 0.001**
Rheumatoid arthritis	15 (1.7)	1 (0.4)	5 (1.7)	0.37
Spondyloarthritis	5 (0.6)	0 (0.0)	0 (0.0)	0.23
Femoral neck fracture	32 (3.6)	22 (9.2)	22 (7.5)	** < 0.001**
Connective tissue disease	0 (0.0)	0 (0.0)	0 (0.0)	–
Autoimmune or systemic disease	66 (7.3)	19 (8.0)	27 (9.1)	
Hypothyroidism	32 (3.6)	11 (4.6)	13 (4.4)	0.61
Hyperthyroidism	4 (0.4)	3 (1.3)	3 (1.0)	0.68
Type 1 diabetes	1 (0.1)	0 (0.0)	0 (0.0)	0.31
Rheumatoid arthritis	14 (1.7)	1 (0.4)	5 (1.7)	0.73
Psoriasis	16 (1.8)	1 (0.4)	6 (2.0)	0.37
Crohn’s disease	2 (0.2)	4 (1.7)	2 (0.7)	0.27
Multiple sclerosis	3 (0.3)	0 (0.0)	0 (0.0)	**0.03**
Systemic disease	11 (1.2)	3 (1.3)	6 (2.0)	0.40
Dysthyroidism	112 (12.5)	43 (18.0)	45 (15.2)	0.07
Hypothyroidism	98 (10.9)	40 (16.7)	38 (12.8)	**0.048**
Hyperthyroidism	14 (1.6)	3 (1.3)	7 (2.4)	0.55
Digestive tract disease				
Digestive ulcer	32 (3.6)	14 (5.9)	18 (6.1)	0.10
Liver disease	29 (3.2)	7 (2.9)	8 (2.7)	0.89
Steatosis	3 (0.3)	1 (0.4)	3 (1.0)	0.34
Cirrhosis	12 (1.3)	4 (1.7)	1 (0.3)	0.29
Viral hepatitis	18 (2.0)	2 (0.8)	4 (1.4)	0.41
Hepatic failure	0 (0.0)	1 (0.4)	0 (0.0)	0.08
Venous thrombotic event	94 (10.5)	32 (13.4)	49 (16.6)	**0.02**
Pulmonary embolism	37 (4.1)	11 (4.6)	18 (6.1)	0.38
Deep vein thrombosis	63 (7.0)	25 (10.5)	42 (14.2)	** < 0.001**
Cerebral venous thrombosis	0 (0.0)	1 (0.4)	0 (0.0)	0.08
Other sites	14 (1.6)	2 (0.4)	6 (2.0)	0.54
Chronic anemia	138 (15.4)	48 (20.1)	83 (28.2)	** < 0.001**
HIV	4 (0.4)	0 (0.0)	1 (0.3)	0.58
Epilepsy	33 (3.6)	19 (7.9)	24 (8.1)	**0.002**
Parkinsonism	10 (1.1)	7 (2.9)	30 (10.2)	** < 0.001**
Psychiatric disorder				
Mood disorder	121 (13.5)	49 (20.5)	76 (25.8)	** < 0.001**
Untreated	32 (3.6)	11 (4.6)	15 (5.1)	0.46
Treated	89 (9.9)	38 (15.9)	60 (20.3)	** < 0.001**
Psychotic disorder	11 (1.2)	9 (3.8)	19 (6.4)	** < 0.001**
Living in an institution	22 (2.4)	33 (13.8)	124 (41.8)	** < 0.001**
Prestroke medications				
Antiplatelet agent	289 (31.9)	89 (36.9)	116 (39.06)	**0.047**
Anticoagulant	178 (19.6)	79 (32.8)	80 (26.9)	** < 0.001**
Antihypertensive treatment	621 (68.5)	181 (75.1)	204 (68.7)	0.13
Antidiabetic	159 (17.5)	50 (20.8)	50 (16.8)	0.44
Lipid-lowering therapy	302 (33.3)	68 (28.2)	73 (24.6)	**0.01**
Stroke type				0.10
Ischemic stroke	808 (89.1)	207 (85.9)	252 (84.9)	
Intracerebral hemorrhage	99 (10.9)	34 (14.1)	45 (15.2)	
NIHSS score at admission, mean (SD)	7.3 (± 8.5)	7.0 (± 7.2)	9.3 (± 8.3)	** < 0.001**
NIHSS score at admission, median (IQR)	4(2–10)	4 (2–10)	7 (2–15)	** < 0.001**
Acute revascularization therapy*				** < 0.001**
None	643 (79.5)	180 (86.9)	238 (94.5)	
Intravenous thrombolysis alone	75 (9.3)	11 (5.3)	6 (2.4)	
Mechanical thrombectomy alone	61 (7.6)	13 (6.3)	5 (1.9)	
Combined treatment	29 (3.6)	3 (1.5)	3 (1.2)	
Hospitalization for stroke	857 (94.8)	232 (96.3)	238 (80.4)	** < 0.001**
Intensive care unit	451 (53.3)	93 (40.6)	53 (22.3)	** < 0.001**
Causes of cognitive impairment				** < 0.001**
Alzheimer’s disease	-	5 (2.1)	53 (17.8)	
Vascular dementia	-	30 (12.5)	45 (15.2)	
Mixed dementia	-	9 (3.7)	51 (17.2)	
Parkinson’s disease	-	1 (0.4)	11 (3.7)	
Other	-	14 (5.8)	23 (7.7)	
Undermined/missing	-	182 (75.5)	114 (38.4)	

Bold indicates *p* < 0.05

*HIV* human immunodeficiency viruses, *NIHSS* National Institutes of Health Stroke Scale, *TIA* transient ischemic attack

^*^Calculated among patients with ischemic stroke (1286 patients)

**Fig. 3:**
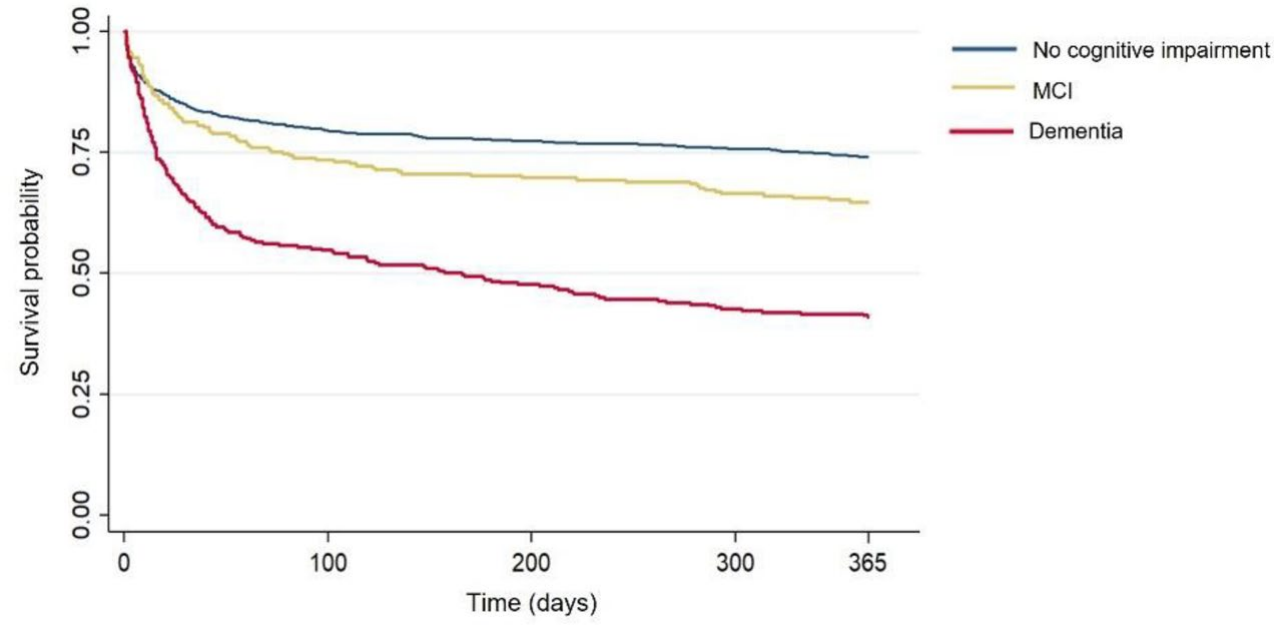
Kaplan–Meier estimates of 1-year post-stroke survival according to prestroke cognitive status. *MCI:* mild cognitive impairment

Overall case-fatality at 1 year was 35.2% and was greater in patients with preexisting dementia (59.3%) than in patients with prior MCI (35.3%) or those without premorbid cognitive impairment (25.7%) (Fig. 3, log-rank test *p* < 0.001). In multivariable models, preexisting dementia was associated with 1-year death (adjusted HR = 1.98; 95% CI 1.60–2.48, *p* < 0.001), while the association was no longer significant for MCI (adjusted HR = 1.11; 95% CI 0.85–1.45, *p* = 0.44) (Table 2). Similar results were observed among patients with ischemic stroke (*n* = 1286) after considering acute revascularization therapy in multivariable models (adjusted HR = 1.98; 95% CI 1.60–2.48, *p* < 0.001 for preexisting dementia and adjusted HR = 1.11; 95% CI 0.85–1.45, *p* = 0.44 for preexisting MCI, Supplemental Table S1).

**Table 2:** Association between prestroke cognitive status and death at 1 year

	HR	(95% CI)	*p*
Unadjusted model			
No cognitive impairment	Ref.	–	–
MCI	1.42	(1.11–1.82)	0.01
Dementia	2.84	(2.33–3.46)	** < 0.001**
Model 1*			
No cognitive impairment	Ref.	–	–
MCI	1.08	(0.84–1.39)	0.56
Dementia	2.04	(1.66–2.52)	** < 0.001**
Model 2**			
No cognitive impairment	Ref.	–	–
MCI	1.11	(0.85–1.45)	0.44
Dementia	1.98	(1.60–2.48)	** < 0.001**

Bold indicates *p* < 0.05

*MCI* mild cognitive impairment

^*^Adjusted for sex and age

^**^Final model with stepwise backward selection adjusted for sex, age, atrial fibrillation, peripheral artery disease, kidney function, hepatic disease, solid cancer, chronic anemia, NIHSS score at admission, and stroke type

**Fig. 4:**
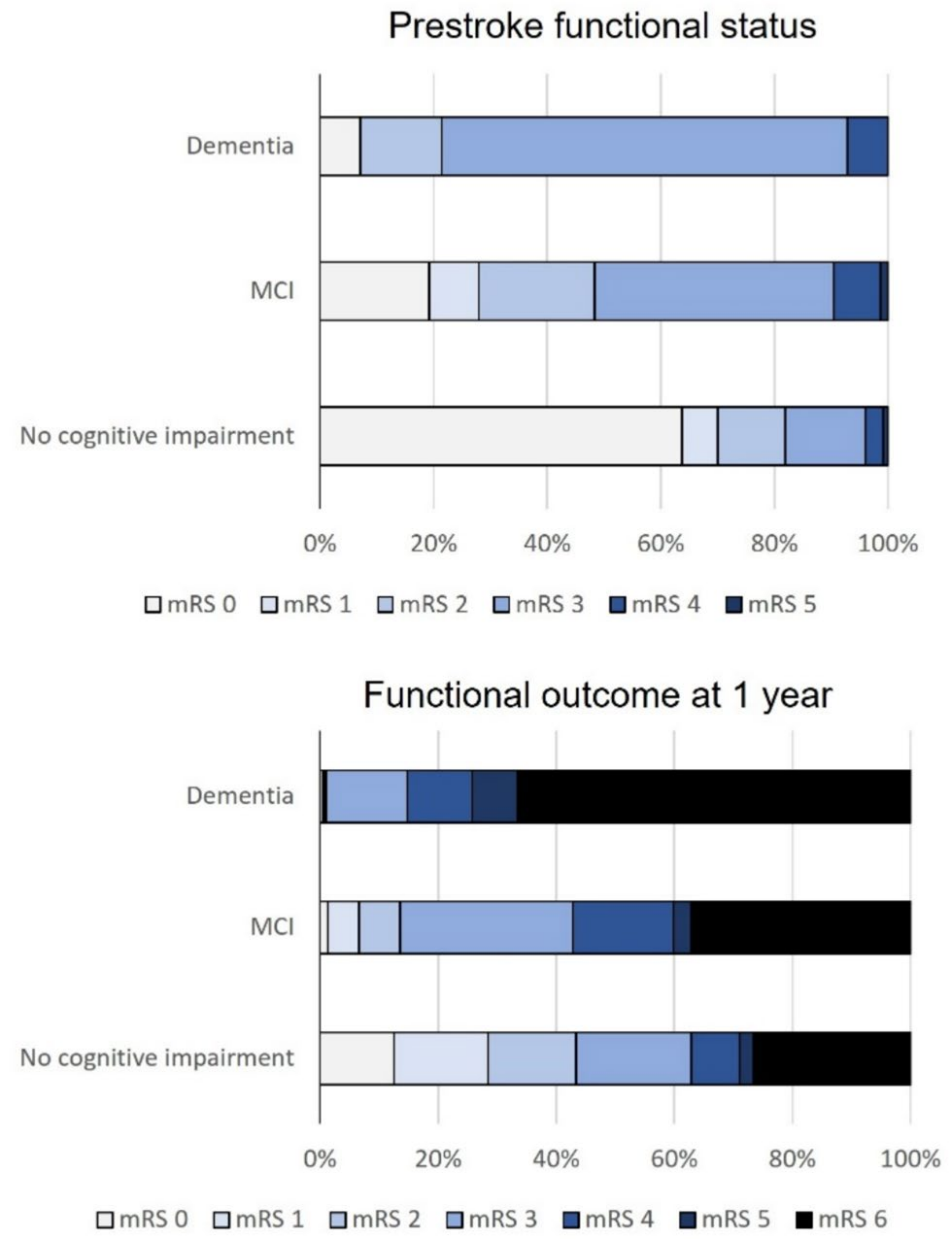
Distribution of prestroke and 1-year mRS score according to prestroke cognitive status. *MCI* mild cognitive impairment, *mRS* modified Rankin Scale

Data regarding mRS score at 1 year were available in 96.5% of patients, with no difference in the proportion of missing information according to prestroke cognitive status. The mRS score was assessed by telephone in 92.2% of cases and was obtained from medical records in 7.8% of cases. There was no difference in the proportion of patients for whom the score was assessed by telephone versus medical record review according to prestroke cognitive status (telephone assessment in 91.5% of patients without cognitive impairment, 92.6% of patients with MCI, and 94.1% of patients with dementia; *p* = 0.35). There were gradually more patients with functional impairment defined by higher mRS score among patients with MCI and those with dementia compared with patients without cognitive impairment (Fig. 4). An unfavorable functional outcome was observed in 49.9% of patients with normal cognition, 66.2% of patients with pretexting MCI, and 81.4% of patients with prestroke dementia. In univariate logistic regression analysis, patients with prior MCI (OR: 1.97; 95% CI 1.45–2.67, *p* < 0.001) and those with preexisting dementia (OR: 4.39; 95% CI 3.17–6.08, *p* < 0.001) had poorer functional outcomes than cognitively not-impaired patients (Table 3). After adjustment for confounding variables including comorbidities, there was a significant association between both MCI (adjusted OR: 1.45; 95% CI 1.01–1.81, *p* = 0.04) and dementia (adjusted OR: 2.65; 95% CI 1.81–3.87, *p* < 0.001), and unfavorable outcome. When analyses were restricted to patients with ischemic stroke, and after adjustment for acute revascularization therapy as a confounder, preexisting dementia remained associated with an unfavorable outcome (adjusted OR = 2.41; 95% CI, 1.61–3.60; *p* < 0.001), whereas the association was not significant for preexisting MCI (adjusted OR = 1.32; 95% CI, 0.91–1.91; *p* = 0.14) (Supplementary Table S2).

**Table 3:** Logistic regression analyses of the association between prestroke cognitive status and unfavorable functional outcome at 1 year

	OR	(95% CI)	*p*
Unadjusted model			
No cognitive impairment	Ref.	–	–
MCI	1.97	(1.45–2.67)	** < 0.001**
Dementia	4.39	(3.17–6.08)	** < 0.001**
Model 1*			
No cognitive impairment	Ref.	–	–
MCI	1.44	(1.04–1.97)	**0.027**
Dementia	2.91	(2.06–4.09)	** < 0.001**
Model 2**			
No cognitive impairment	Ref.	–	–
MCI	1.45	(1.01–1.81)	**0.04**
Dementia	2.65	(1.81–3.87)	** < 0.001**

Bold indicates *p* < 0.05

*MCI* mild cognitive impairment

^*^Adjusted for sex and age

^**^Final model with stepwise backward selection adjusted for sex, age, hypertension, diabetes, kidney function, solid cancer, chronic anemia, NIHSS at admission, antihypertensive treatment, and stroke type

## Discussion

In this population-based stroke cohort, preexisting cognitive impairment was associated with poorer post-stroke outcomes. The observed associations were independent of other major comorbidities.

Previous studies found that stroke patients with prior cognitive impairment have a poorer prognosis [3, 4, 6–9, 16, 24, 25]. Our findings indicated that, compared to patients with normal cognition before stroke, mRS scores and case-fatality rates increased among patients with preexisting MCI and even more so in patients with dementia. Recognizing that the prestroke functional score may reflect more than neurological condition alone, we analyzed changes in mRS from baseline to 1-year post-stroke to classify outcomes as favorable or unfavorable. Our results showed unfavorable functional outcomes in the MCI and dementia groups compared to those with normal cognitive function at baseline. One-year survival was also significantly lower among cognitively impaired patients, with dementia being associated with a twofold increased risk of death. Notably, this association did not reach statistical significance in patients with prior MCI, unlike the association observed for functional outcomes. This discrepancy suggests that some factors may negatively affect long-term functional recovery without directly impacting short-term survival. Poststroke cognitive decline may be a contributing factor, particularly given its known interference with rehabilitation efficacy and functional participation [26]. In our population, a history of cerebrovascular events was significantly more frequent in patients with cognitive impairment. Since chronic cerebrovascular lesions are the second major cause of cognitive impairment in the general population, prestroke MCI may exacerbate cognitive decline after a stroke. In our cohort, a subset of patients may have developed cognitive disorders following a prior stroke but were considered to have prestroke cognitive impairment at baseline. This highlights the complex mechanisms underlying the negative impact of cognitive disorders before or after stroke on prognosis in patients more exposed to recurrence of cerebrovascular events [27–29].

Our study adds to the existing body of knowledge on this topic by taking into account a wide range of patient comorbidities. In a previous multicentric study, patterns of comorbidities were collected in patients with first-ever stroke to analyze their impact on functional and cognitive prognosis, but prestroke cognitive status was not considered [30]. Conversely, there is increasing evidence that prestroke cognitive disorders are associated with higher numbers of comorbidities, reflecting a frailer state [31–33]. A few studies have specifically explored the interactions between cognitive impairment and chronic comorbid conditions in relation to post-stroke outcomes [34]. Our results support the hypothesis that cognitive impairment constitutes an independent prognostic factor when accounting for established risk factors and comorbidities. However, a large observational study from the Canadian Stroke Network Registry reported differing results [35]. Following cohort matching, no significant difference in 1-year survival was observed between patients with and without dementia. However, disability was assessed only at hospital discharge, which may not fully capture long-term outcomes. Additionally, the cohorts were not fully comparable, as our study included both ischemic and hemorrhagic strokes, and the methods of adjustment differed. Comorbidities in the Canadian study were assessed using a global index (Charlson Comorbidity Score), whereas our analyses considered each comorbid condition individually. Further studies using standardized definitions and adjustment strategies are required to obtain reproducible results.

In line with previous observational studies focusing on ischemic stroke, there were lower rates of acute revascularization in cognitively impaired groups, although there are no formal recommendations regarding cognitive status [36]. These figures may suggest a certain reluctance among physicians to pursue invasive procedures given the expected risks and uncertain benefits, especially in older adults [37, 38]. However, analyses restricted to patients with ischemic stroke suggest that differences in the use of revascularization therapies between groups did not explain the higher mortality and poorer functional outcomes observed in patients with preexisting dementia. In contrast, among patients with preexisting mild cognitive impairment (MCI), the association with unfavorable outcome was no longer statistically significant after adjustment for acute revascularization therapies; however, a lack of statistical power cannot be excluded.

There is, therefore, a need to better understand the interactions between frailty and stroke, conditions that frequently coexist [1]. In our study, we approached frailty through the lens of cognitive impairments and multimorbidity. However, we acknowledge that frailty is a wider concept that should be assessed through a broader range of clinical, functional, and biological indicators. Nonetheless, including these patients in prospective studies with a high level of evidence is needed to support decision-making.

Our study has several strengths. The population-based design allowed for a comprehensive collection of stroke cases, regardless of their management, thanks to multiple overlapping sources of information. The large sample size and longitudinal follow-up with minimal missing data enabled multivariable analyses. However, some limitations must be acknowledged. Although several sources of information were collected to assess prestroke cognitive status, there was no formal cognitive objective evaluation [16]. Identification of prestroke cognitive impairment is not homogenous in the literature. Several studies used the Informant Questionnaire on Cognitive Decline in the Elderly (IQCODE) [8, 39], which was not possible here due to our population-based approach. However, in our cohort, 37% of patients had preexisting cognitive impairment, which is in line with estimations given in the literature regarding stroke population [27]. The absence of systematic post-stroke cognitive assessments precluded evaluation of the dynamic interaction between preexisting and incident cognitive decline. In addition, given the population-based design of the study, the etiology of preexisting dementia or mild cognitive impairment (MCI) was frequently undetermined. This likely reflects the limited diagnostic investigations performed in routine clinical practice for a substantial proportion of patients and prevented the conduct of etiological subgroup analyses. Cause-specific mortality was not available in the national death registry used for follow-up. Therefore, we could not distinguish between stroke-related death, dementia progression, recurrent events, or other causes, which limited analyses assessing the causal impact of comorbidities on patient survival.

## Conclusion

Prestroke dementia was associated with significantly poorer functional outcomes and increased mortality within the first year following stroke. To a lesser extent, MCI also appeared to predict reduced functional recovery. These associations persisted after adjustment for established prognostic factors and comorbidities, highlighting the independent contribution of cognitive status to post-stroke prognosis. The observed gradient of outcomes across cognitive impairment levels emphasizes the importance of early assessment in the acute phase of stroke care. Given the growing prevalence of cognitive disorders in the aging population, this profile of patients should be included in larger clinical studies in order to develop adapted and ethical approaches to care.

## Supplementary Information

Below is the link to the electronic supplementary material.

Supplementary file1 (DOCX 24 KB)

## Data Availability

All data generated or analyzed during this study are included in this published article.
