# Supplementary material for: Influence of preexisting cognitive impairment and comorbidities on post-stroke outcomes: Dijon Stroke Registry
Source: J Neurol. 2026 Apr 1;273(4):241. doi: 10.1007/s00415-026-13782-5 (PMC13043513; doi:10.1007/s00415-026-13782-5)
Supplement: Supplementary file 1 — Supplementary file1 (DOCX 24 KB) [file 415_2026_13782_MOESM1_ESM.docx]

**Supplementary File**

**Supplemental methods**

The following chronic conditions were collected: chronic pulmonary disease (chronic obstructive pulmonary disease, asthma, restrictive ventilatory defect), rheumatologic disease (osteoporosis, rheumatoid polyarthritis, spondyloarthritis), femoral neck fracture, pathology of conjunctive tissue, systemic and autoimmune pathologies (thyroid, type 1 diabetes, rheumatoid polyarthritis, psoriasis, Crohn’s disease, multiple sclerosis), dysthyroidism (hypothyroidism or hyperthyroidism including non-autoimmune etiologies), digestive ulcer, liver disease (steatosis, cirrhosis, viral chronic hepatitis, liver failure), kidney function (classified in five stages according to CKD-EPI clearance calculation : 0 - normal kidney function, 1- clearance 60-90ml/min, 2-clearance 30-59 ml/min, 3-clearance 15-29 ml/min, 4-clearance <15ml/min or extrarenal epuration), solid cancer (no history of cancer, cured cancer, localized, lymph node metastasis, organic metastasis), hemopathy (lymphoma, chronic lymphoid leukemia, acute leukemia, myeloma), venous thromboembolic event (pulmonary embolism, deep vein thrombosis, venous cerebral thrombosis, other localization), chronic anemia (defined by hemoglobin rate ≤13 dg/L in males, ≤ 12dg/L in females, for at least 6 month or during several hospital stays), human immunodeficiency virus (HIV) positive status, epilepsy, Parkinsonism, mood disorder (treated or untreated at the time of event), psychotic condition.

**Supplemental Table S1:** Association between prestroke cognitive status and death at one year in patients with ischemic stroke

|  | **HR** | **(95% CI)** | **p** |
| --- | --- | --- | --- |
| Unadjusted model |  |  |  |
| No cognitive impairment | *Ref.* | - | - |
| MCI | 1.39 | (1.05-1.84) | 0.02 |
| Dementia | 3.09 | (2.49-3.84) | **<0.001** |
| Model 1* |  |  |  |
| No cognitive impairment | *Ref.* | - | - |
| MCI | 1.04 | (0.78-1.38) | 0.81 |
| Dementia | 2.16 | (1.72-2.72) | **<0.001** |
| Model 2** |  |  |  |
| No cognitive impairment | *Ref.* | - | - |
| MCI | 1.09 | (0.80-1.48) | 0.58 |
| Dementia | 1.89 | (1.47-2.43) | **<0.001** |

* *Adjusted for sex and age*

*** Final model with stepwise backward selection adjusted for sex, age, atrial fibrillation, diabetes, kidney function, hepatic disease, solid cancer, chronic anemia, psychotic condition, NIHSS score at admission, acute revascularization therapy*

*MCI: mild cognitive impairment*

**Supplemental Table S2:** Logistic regression analyses of the association between prestroke cognitive status and unfavorable functional outcome at one year in patients with ischemic stroke.

|  | **OR** | **(95% CI)** | **p** |
| --- | --- | --- | --- |
| Unadjusted model |  |  |  |
| No cognitive impairment | *Ref.* | - | - |
| MCI | 1.93 | (1.40-2.67) | **<0.001** |
| Dementia | 4.51 | (3.19-6.38) | **<0.001** |
| Model 1* |  |  |  |
| No cognitive impairment | *Ref.* | - | - |
| MCI | 1.39 | (0.99-1.95) | 0.06 |
| Dementia | 2.92 | (2.03-4.21) | **<0.001** |
| Model 2** |  |  |  |
| No cognitive impairment | *Ref.* | - | - |
| MCI | 1.32 | (0.91-1.91) | **0.14** |
| Dementia | 2.41 | (1.61-3.60) | **<0.001** |

* *Adjusted for sex and age*

***Final model with stepwise backward selection adjusted for sex, age, diabetes, peripheral artery disease, solid cancer, chronic anemia, NIHSS at admission, acute revascularization therapy*

*MCI: mild cognitive impairment*
